# Predictors of influenza a molecular viral shedding in Hutterite communities

**DOI:** 10.1111/irv.12448

**Published:** 2017-03-16

**Authors:** Biao Wang, Margaret L. Russell, Kevin Fonseca, David J. D. Earn, Gregory Horsman, Paul Van Caeseele, Khami Chokani, Mark Vooght, Lorne Babiuk, Stephen D. Walter, Mark Loeb

**Affiliations:** ^1^ Department of Pathology and Molecular MedicineMcMaster UniversityHamiltonONCanada; ^2^ Department of Community Health SciencesCumming School of MedicineUniversity of CalgaryCalgaryABCanada; ^3^ Department of Microbiology and Infectious Diseases and Provincial Laboratory for Public HealthUniversity of CalgaryCalgaryABCanada; ^4^ Department of Clinical Epidemiology and BiostatisticsMcMaster UniversityHamiltonONCanada; ^5^ Michael G. De‐ Groote Institute for Infectious Disease ResearchMcMaster UniversityHamiltonONCanada; ^6^ Department of Mathematics and StatisticsMcMaster UniversityHamiltonONCanada; ^7^ Saskatchewan Disease Control LaboratoryReginaSKCanada; ^8^ Cadham Provincial LaboratoryWinnipegMBCanada; ^9^ Saskatchewan HealthPrince Albert Parkland Health RegionPrince AlbertSKCanada; ^10^ Saskatchewan HealthFive Hills Health RegionMoose JawSKCanada; ^11^ University of AlbertaEdmontonABCanada; ^12^ Department of MedicineMcMaster UniversityHamiltonONCanada

**Keywords:** influenza viral shedding, predictors, viral AUC, viral load, viral shedding duration

## Abstract

**Background:**

Patterns of influenza molecular viral shedding following influenza infection have been well established; predictors of viral shedding however remain uncertain.

**Objectives:**

We sought to determine factors associated with peak molecular viral load, duration of shedding, and viral area under the curve (AUC) in children and adult Hutterite colony members with laboratory‐confirmed influenza.

**Methods:**

A cohort study was conducted in Hutterite colonies in Alberta, Canada. Flocked nasal swabs were collected during three influenza seasons (2007‐2008 to 2009‐2010) from both symptomatic and asymptomatic individuals infected with influenza. Samples were tested by real‐time reverse‐transcription polymerase chain reaction for influenza A and influenza B, and the viral load was determined for influenza A‐positive samples.

**Results:**

For seasonal H1N1, younger age was associated with a larger AUC, female sex was associated with decreased peak viral load and reduced viral shedding duration, while the presence of comorbidity was associated with increased peak viral load. For H3N2, younger age was associated with increased peak viral load and increased AUC. For pandemic H1N1, younger age was associated with increased peak viral load and increased viral AUC, female sex was associated with reduced peak viral load, while inapparent infection was associated with reduced peak viral load, reduced viral shedding duration, and reduced viral AUC.

**Conclusions:**

Patterns of molecular viral shedding vary by age, sex, comorbidity, and the presence of symptoms. Predictor variables vary by influenza A subtype.

## 1 Introduction

Influenza is a major cause of annual epidemics of respiratory illness, with the potential to lead to hospitalizations and death.^1^, ^2^, ^3^ A better knowledge of viral shedding of influenza can improve our understanding of transmission and ultimately help in prevention efforts.^4^ Data however are limited. Most recent studies on influenza viral shedding have focused on the characteristics of 2009 pandemic H1N1 (pH1N1) viral shedding in either community or institutional settings.^5^, ^6^, ^7^, ^8^, ^9^, ^10^, ^11^ Few studies characterize viral shedding patterns following naturally acquired seasonal influenza and pH1N1 and most are only descriptive.^4^, ^12^, ^13^, ^14^ Factors associated with influenza viral loads, duration of viral shedding, and viral area under the curve (AUC) remain uncertain.^15^, ^16^

In our previous study,^17^ we described molecular viral shedding patterns in naturally infected children and adults over multiple seasons in Hutterite communities. The objective of this study was to examine predictors of viral shedding patterns in these participants.

## 2 Methods

### 2.1 Study population and surveillance

The study population and surveillance methods are described in detail in our previous study.^17^ To summarize, children and adults residing in 10 Hutterite colonies within 150 km from the city of Red Deer, Alberta, were enrolled. The surveillance period was from December 29, 2007, until June 15, 2010. All study participants were assessed twice weekly by a research nurse using a standardized self‐reported symptom or sign checklist, which was completed by a representative of a family for all family members and was provided when the research nurse made a site visit. Study participants with any new symptoms reported were interviewed by the research nurse while onsite at the colony. Children aged between 36 months and 15 years in colonies were vaccinated against influenza in the three flu seasons during the study period with inactivated influenza vaccine.

One nasopharyngeal specimen and one flocked nasal specimen were collected from participants who had two or more of the following symptoms: fever (≥38°C), cough, nasal congestion, sore throat, headache, sinus problems, muscle aches, fatigue, ear ache, and chills. Flocked nasal swabs were collected for up to 8 weeks from the participants whose nasopharyngeal swab tested positive for influenza by reverse‐transcription polymerase chain reaction (RT‐PCR). Specimens were collected daily for 7 days and then every two days for up to 8 weeks or when two consecutive specimens tested negative.

We also collected specimens from asymptomatic participants in colonies when it was established that the colonies had an outbreak, which we defined as ≥2 positive specimens within any 48‐hour period. Specimens from asymptomatic participants were collected for up to 3 weeks: daily for the first week and then every second day until two consecutive specimens tested negative or for a maximum of 3 weeks. Molecular viral load for influenza A was determined by Applied Biosystems One‐Step RT‐PCR kit; the amplification targets matrix gene for influenza A. The research protocol was approved by McMaster University Research Ethics Review Board and by the Conjoint Health Research Ethics Board of the University of Calgary; written informed consent was obtained from all participants.

### 2.2 Statistical analysis

We performed descriptive statistical analyses to summarize the characteristics by age, sex, comorbidity, and vaccination status of the study participants over the three seasons. We also used mixed‐effect univariate logistic regression to test the association between these predictors and the development of influenza.

We then analyzed factors associated with peak viral load, duration of viral shedding, and viral AUC in this study. Factors considered in our analyses were age, sex, comorbidity, vaccination status, and asymptomatic status. Univariate analysis was performed using Student's *t* test. Factors with a *P*‐value <.2 in univariate analysis were considered for multivariable analysis. We used backwards selection method to build our final models and retained variables with a *P*‐value <.05. For all multivariable analyses, we used mixed‐effect models to account for the hierarchical nature of the data (ie, the effect of colony and household).^18^, ^19^

We defined peak viral load (expressed in copies/mL and then log‐transformed) as the maximum viral RNA concentrations in all specimens collected from a participant during an episode of infection and used mixed‐effect regression models to identify independent predictors. An episode of infection began upon the initial detection of influenza by RT‐PCR and ended when two consecutive RT‐PCR tests were negative. We defined prolonged shedding as detectable viral RNA at >5 days after the episode start date.^20^ We analyzed duration of viral shedding using mixed‐effect Cox proportional hazard. Viral AUC was calculated to assess both the level and duration of viral shedding with a single parameter [20]. We adopted the definition of viral AUC from Shaefer et al.^16^ as in Equation (1).

(1) $$\text{AU}C_{n}=\frac{1}{2}\sum_{i=1}^{n-1}\left(t_{i+1}-t_{i}\right)\left(y_{i+1}+y_{i}\right),\;\;\;\;\;\;\;\;\;\;\;\;\;\;\;\;\;n\geq 2$$

where *t*_1_,*t*_2_,…,*t*_*n*_ were the days on which samples were collected; *y*_1_, *y*_2_,…..,*y*_*n*_ were corresponding viral concentration levels measured on the days. Viral AUC (expressed in copies/mL‐days and then log‐transformed) was modeled using mixed‐effect regression models.

In all analyses, a *P*‐value of <.05 was considered to indicate statistical significance. All probabilities were two‐tailed. Statistical analyses were performed with R (version 3.2.2) software.^21^

## 3 Results

### 3.1 Baseline characteristics

839 people in 194 households from 10 Hutterite colonies participated in the study over 3 years. There were 393 participants in 2007‐2008, 776 in 2008‐2009, and 745 in 2009‐2010 for a total of 1,914 participant‐seasons. Characteristics of study participants over the study period were similar (Table 1).

**Table 1:** Baseline characteristics of participants over the three seasons

	2007‐08 season N=393	2008‐09 season N=776	2009‐10 season N=745
	No. (%)	No. (%)	No. (%)
Age (≥16 y)	152 (39)	404 (52)	393 (53)
Sex (male)	170 (43)	344 (44)	324 (44)
≥ 1 comorbidity^a^	44 (11)	67 (9)	61 (8)
Vaccination	191 (49)	263 (34)	253 (36)

a ≥1 comorbidity refers to the presence of one or more of the following illness: heart or lung disorders, blood disorders, swallowing or choking problems, chronic metabolic diseases, kidney or liver diseases, cancer, immunodeficiency, immunosuppression, and conditions that require treatment for long periods with acetylsalicylic acid.

There were 195 episodes of RT‐PCR‐confirmed influenza A infection observed over the three seasons. Of these, 62 were seasonal influenza H1N1 (A/Solomon Islands/3/2006 H1N1‐like) detected in 2007‐2008; 36 were seasonal influenza H3N2 (A/Brisbane/10/2007 H3N2‐like) detected in 2008‐2009; and 97 cases were pandemic 2009 H1N1 in 2009‐2010 season.

**Figure 1:**
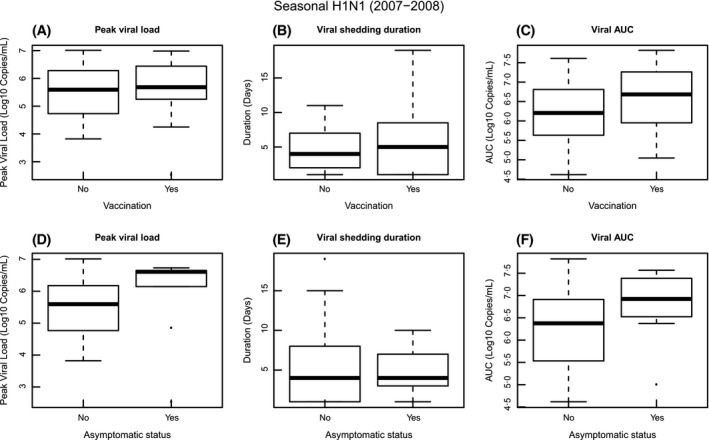
Comparison of peak viral load, viral shedding duration, and viral AUC for seasonal H1N1 between non‐vaccinated and vaccinated cases and between asymptomatic and symptomatic cases. A, peak viral load comparison between non‐vaccinated and vaccinated cases; B, viral shedding duration comparison between non‐vaccinated and vaccinated cases; C, viral AUC comparison between non‐vaccinated and vaccinated cases; D, peak viral load comparison between asymptomatic and symptomatic cases; E, viral shedding duration comparison between asymptomatic and symptomatic cases; F, viral AUC comparison between asymptomatic and symptomatic cases

Nine (15%) of the 62 seasonal H1N1 cases were asymptomatic (Table 2). Of these, seven (78%, 7/9) cases had other seasonal H1N1 cases in their household. 31 (50%) of the 62 seasonal H1N1 cases had received the seasonal vaccine in 2007‐2008 season (Table 2). Of these, 26 (84%, 26/31) cases had other seasonal H1N1 influenza cases in their household. There were six seasonal H1N1 cases that were both asymptomatic and vaccinated with the seasonal vaccine. Of these, two (66%, 4/6) had other seasonal H1N1 cases in their household. Figure 1 compares peak viral load, viral shedding duration, and viral AUC for seasonal H1N1 between asymptomatic and symptomatic cases and between non‐vaccinated and vaccinated cases.

**Table 2:** Characteristics of influenza A infection cases over the three seasons

	No (%)	OR (95% CI)	*P*
2007‐08 season H1N1 (N=62)			
Age (≥ 16 y)	19 (30)	0.74 (0.33‐1.66)	.47
Sex (male)	28(45)	1.06 (0.51‐2.22)	.87
≥1 comorbidity	10 (16)	1.43 (0.48‐4.24)	.52
Vaccination	31 (50)	0.94 (0.44‐2.02)	.88
Asymptomatic	9 (15)	–	–
2008‐09 season H3N2 (N=36)			
Age (≥16 y)	19 (53)	1.15 (0.36‐3.63)	.82
Sex (male)	13(36)	0.58 (0.22‐1.58)	.29
≥1 comorbidity	1 (3)	1.07 (0.09‐12.98)	.96
Vaccination	5 (14)	0.11 (0.03‐0.45)	<.01
Asymptomatic	0 (0)	–	–
2009‐10 season pH1N1 2009 (N=97)			
Age (≥ 16 y)	24 (25)	0.16 (0.08‐0.31)	<.01
Sex (male)	43(44)	0.79 (0.45‐1.37)	.40
≥1 comorbidity	5 (5)	0.68 (0.22‐2.16)	.51
Vaccination	58 (60)	6.11 (3.11‐12.01)	<.01
Asymptomatic	12 (12)	–	–

pH1N1, pandemic H1N1; OR, odds ratio; CI, confidence interval.

**Figure 2:**
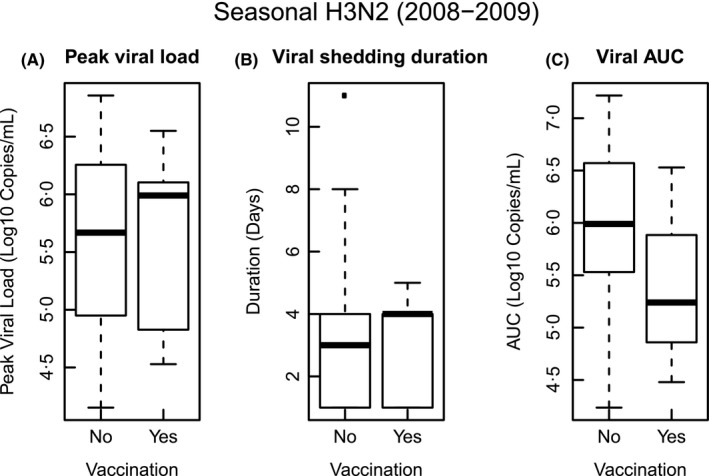
Comparison of peak viral load, viral shedding duration, and viral AUC for seasonal H3N2 between non‐vaccinated and vaccinated cases. A, peak viral load comparison between non‐vaccinated and vaccinated cases; B, viral shedding duration comparison between non‐vaccinated and vaccinated cases; C, viral AUC comparison between non‐vaccinated and vaccinated cases

There were no asymptomatic seasonal H3N2 cases (Table 2). Five (14%) of the 36 seasonal H3N2 cases had received the seasonal vaccine in 2008‐2009 season (Table 2). Of these, two (40%, 2/5) cases had other seasonal H3N2 cases in their household. Figure 2 compares peak viral load, viral shedding duration, and viral AUC for seasonal H3N2 between non‐vaccinated and vaccinated cases.

**Figure 3:**
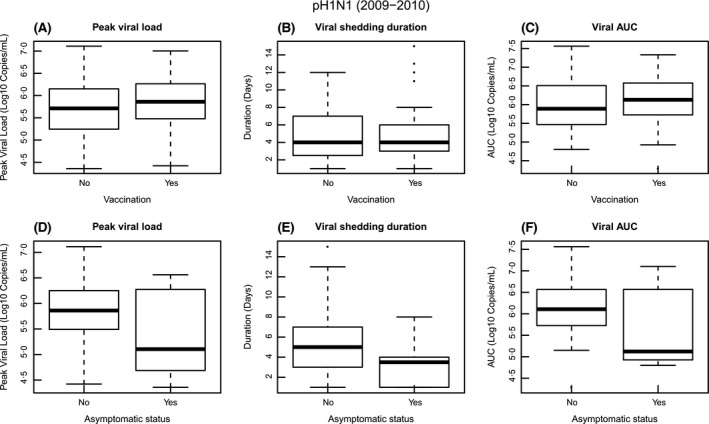
Comparison of peak viral load, viral shedding duration, and viral AUC for pH1N1 between asymptomatic and symptomatic cases and between non‐vaccinated and vaccinated cases. A, peak viral load comparison between non‐vaccinated and vaccinated cases; B, viral shedding duration comparison between non‐vaccinated and vaccinated cases; C, viral AUC comparison between non‐vaccinated and vaccinated cases; D, peak viral load comparison between asymptomatic and symptomatic cases; E, viral shedding duration comparison between asymptomatic and symptomatic cases; F, viral AUC comparison between asymptomatic and symptomatic cases

12 (12%) of the 97 pandemic H1N1 cases were asymptomatic (Table 2). Of these, seven (58%, 7/9) cases had other influenza cases in their household. 58 (60%) of the 97 pandemic H1N1 cases had received the seasonal vaccine in 2009‐2010 season (Table 2). Of these, 52 (90%, 52/58) cases had other influenza cases in their household. Figure 3 compares peak viral load, viral shedding duration, and viral AUC for seasonal H1N1 between asymptomatic and symptomatic cases and between non‐vaccinated and vaccinated cases.

Two (4%, 2/53) symptomatic seasonal H1N1 cases were detected to have pre‐symptomatic shedding; one had shedding starting 1 day before and the other 2 days before symptom onset; one (3%, 1/36) symptomatic seasonal H3N2 case was detected to have pre‐symptomatic shedding starting 1 day before symptom onset; five (6%, 5/85) symptomatic pH1N1 cases were detected to have pre‐symptomatic shedding, with a shedding start date distributed as 1(20%), 3(40%), 4(20%), 5 (20%) days before symptom onset.

As shown in Table 2, older age was significantly associated with lower risk for pH1N1 infection (OR 0.16, 95% CI 0.08 to 0.31). In addition, vaccination with the 2008/2009 seasonal vaccine was significantly associated with lower risk for H3N2 infection (OR 0.11, 95% CI 0.03 to 0.45), whereas vaccination with the 2009/2010 seasonal vaccine was significantly associated with higher risk for pH1N1 infection (OR 6.11, 95% CI 3.11 to 12.01).

### 3.2 Peak load analysis

For the 2007‐2008 season (seasonal H1N1), we found that the mean peak viral load (±SD) among males was significantly greater than in females (5.87±0.96 vs 5.37±0.85 log_10_ copies/mL, *P*=.04) (Table 3) in univariate analysis. Age (*P*=.09) and comorbidity (*P*=.08) (Table 3), along with sex, were entered into multivariate analysis. The final model showed female sex (β, −0.38; 95% CI, −0.71 to −0.04; *P* = .04) to be associated with a decreased peak viral load while the presence of comorbidity (β, 0.72; 95% CI, 0.26 to 1.28; *P*<.01) was associated with an increased peak viral load for seasonal H1N1 (Table 4).

**Table 3:** Peak viral load, viral shedding duration, and viral AUC compared between participants with different characteristics

	Peak viral load, log_10_ Copies/mL		Viral shedding duration, days		Viral AUC, log_10_ Copies/mL‐days	
	mean ± SD	*P*	mean ± SD	*P*	mean ± SD	*P*
2007‐08 season (H1N1)						
Age						
≤15 y	5.73±0.96	.09	5.53±4.37	.30	6.62±0.79	<.01
≥16 y	5.31±0.81		4.52±3.07		5.86±0.76	
Sex						
Female	5.37±0.85	.04	4.29±3.83	.04	6.16±0.93	.11
Male	5.87±0.96		6.36±4.01		6.57±0.74	
Comorbidity						
Any	6.03±0.76	.08	4.90±3.28	.75	6.64±0.72	.17
No	5.52±0.95		5.28±4.17		6.33±0.87	
Vaccination						
Yes	5.66±0.95	.58	5.77±4.71	.29	6.56±0.80	.18
No	5.53±0.92		4.67±3.17		6.22±0.87	
Asymptomatic						
No	5.54±0.83	.44	5.26±4.19	.82	6.78±0.83	.17
Yes	5.92±1.40		5.00±3.00		6.30±0.84	
2008‐09 season (H3N2)						
Age						
≤15 y	6.05±0.70	<.01	4.18±2.79	.07	6.32±0.82	<.01
≥16 y	5.26±0.60		2.68±1.89		5.45±0.72	
Sex						
Female	5.50±0.68	.20	2.69±1.72	.05	5.72±0.87	.20
Male	5.86±0.84		4.62±3.07		6.18±0.85	
Comorbidity						
Any	–	–	–	–	–	–
No	5.67±0.72		3.46±2.44		5.90±0.87	
Vaccination						
Yes	5.60±0.87	.93	3.0±1.87	.65	5.41±1.04	.45
No	5.64±0.75		3.45±2.54		5.97±0.86	
Asymptomatic						
No	5.63±0.75	–	3.38±2.44	–	5.90±0.87	–
Yes	–		–		–	
2009‐10 season (pH1N1 2009)						
Age						
≤15 y	5.94±0.59	<.01	5.00±3.02	.10	6.24±0.68	<.01
≥16 y	5.44±0.52		4.00±2.38		5.61±0.43	
Sex						
Female	5.64±0.67	<.01	4.70±2.90	.85	5.95±0.75	.02
Male	6.03±0.45		4.81±2.91		6.28±0.53	
Comorbidity						
Any	5.73±0.41	.66	5.20±3.00	.75	5.99±0.41	.64
No	5.82±0.62		4.73±2.90		6.10±0.69	
Vaccination						
Yes	5.87±0.59	.26	4.84±2.88	.72	6.13±0.65	.59
No	5.72±0.64		4.61±2.94		6.04±0.73	
Asymptomatic						
No	5.88±0.55	.05	4.97±2.93	.02	6.15±0.63	.04
Yes	5.35±0.82		3.16±2.12		5.64±0.94	

pH1N1, pandemic H1N1; AUC; area under the curve.

Comorbidity (Any) refers to the presence of one or more of the following illness: heart or lung disorders, blood disorders, swallowing or choking problems, chronic metabolic diseases, kidney or liver diseases, cancer, immunodeficiency, immunosuppression, and conditions that require treatment for long periods with acetylsalicylic acid.

**Table 4:** Explanatory variables in final models for peak viral load, viral shedding duration, and viral AUC^a^

	Peak viral Load		Viral Shedding Duration^b^		Viral AUC	
	β (95% CI)	*P*	HR (95% CI)	*P*	β (95% CI)	*P*
2007‐08 season (H1N1)						
Age (≤15 y vs ≥16 y)	–	–	–	–	0.75 (0.34‐1.16)	<.01
Sex (Female vs Male)	−0.38 (−0.71‐−0.04)	.04	2.46 (1.19‐5.10)	.02	–	–
Comorbidity (Any vs No)	0.72 (0.26‐1.18)	<.01	–	–	–	–
Vaccination (Yes vs No)	–	–	–	–	–	–
Asymptomatic (No vs Yes)	–	–	–	–	–	–
2008‐09 season (H3N2)						
Age (≤15 y vs ≥16 y)	0.79 (0.36‐1.21)	<.01	–	–	0.87 (0.26‐1.48)	.02
Sex (Female vs Male)	–	–	–	–	–	–
Comorbidity (Any vs No)	–	–	–	–	–	–
Vaccination (Yes vs No)	–	–	–	–	–	–
Asymptomatic (No vs Yes)	–	–	–	–	–	–
2009‐10 season (pH1N1 2009)						
Age (≤15 y vs ≥16 y)	0.42 (0.18‐0.66)	<.01	–	–	0.62 (0.31‐0.92)	<.01
Sex (Female vs Male)	−0.30 (−0.51‐−0.10)	<.01	–	–	–	–
Comorbidity (Any vs No)	–	–	–	–	–	–
Vaccination (Yes vs No)	–	–	–	–	–	–
Asymptomatic (No vs Yes)	0.48 (0.16‐0.79)	<.01	0.23 (0.10‐0.58)	<.01	0.55 (0.11‐0.98)	.02

pH1N1, pandemic H1N1; AUC, area under the curve; HR, hazard ratio; CI, confidence interval; β, regression coefficient.

a For the analysis of peak viral load and viral AUC, we used mixed‐effect regression model to account for the hierarchy structure of the data (colony and household); for the analysis of viral shedding duration, we used mixed‐effect Cox proportional hazards regression model to account for the hierarchy structure of the data set.

b We defined prolonged shedding as detectable viral RNA at>5 d after the episode start date.

For 2008‐2009 season (H3N2), we found that the mean peak viral load (±SD) among younger cases (≤15 years) was significantly greater than in older cases (≥16 years) (6.05±0.70 vs 5.26±0.60 log10 copies/mL, *P*<.01) (Table 3) in univariate analysis. Age was the only variable entered into multivariate analysis model. The final model multivariable analysis suggested that younger age (β, 0.79; 95% CI, 0.36 to 1.21 to −0.36; *P*<.01) was associated with an increased peak viral load for H3N2 (Table 4).

For the 2009‐2010 season (pH1N1 2009), we found that the mean peak viral load (±SD) among younger cases (≤15 years) was significantly greater than in older cases (≥16 years) (5.94±0.59 vs 5.44±0.52 log_10_ copies/mL, *P*<.01) and the mean peak viral load (±SD) among males was significantly greater than in females (6.03±0.45 vs 5.64±0.67 log_10_ copies/mL, *P*<.01) (Table 3) in univariate analysis. Asymptomatic status (*P*=.05) was entered into multivariate analysis as was age and sex. The final multivariable model showed younger age (β, 0.42; 95% CI, 0.18 to 0.66; *P*<.01) and being symptomatic (β, 0.48; 95% CI, 0.16 to 0.79; *P*<.01) were associated with an increased peak viral load, while female sex (β, −0.30; 95% CI, −0.51 to −0.10; *P*<.01) was associated with a decreased peak viral load for pH1N1 (Table 4).

### 3.3 Duration of viral Shedding

For the 2007‐2008 season (seasonal H1N1), we found that mean viral shedding duration (±SD) among males was significantly longer than in females (6.36±4.01 vs 4.29±3.83 days, *P*=.04) (Table 3) in univariate analysis. Sex, with a *P*‐value <.2 in univariate analysis, was the only variable entered into multivariable analysis model. The final model from multivariate analysis suggested that female sex (HR, 2.46; 95% CI, 1.19 to 5.10; *P* = .02) was associated with a shorter viral shedding duration for seasonal H1N1 (Table 4).

For the 2008‐2009 season (H3N2), we found that the mean viral shedding duration (±SD) among males was significantly longer than in females (4.62±3.07 vs 2.69±1.72 days, *P*=.05) (Table 3) in univariate analysis. Age was entered into the multivariate analysis model along with sex. The final multivariable model revealed none of the variables tested to be associated with prolonged viral shedding for H3N2.

For 2009‐2010 season (pH1N1 2009), we found mean viral shedding duration (±SD) among symptomatic cases to be significantly longer than in asymptomatic cases (4.97±2.93 vs 3.16±2.12 days, *P*=.02) (Table 3). Age (*P*=.10) was entered into the multivariable model as well as asymptomatic status. The final model analysis demonstrated that being symptomatic (HR, 0.24; 95% CI, 0.10 to 0.58; *P*<.01) was associated with prolonged viral shedding duration for pH1N1 (Table 4).

### 3.4 Viral AUC Analysis

Viral AUC values were calculated for the cases who had more than one viral load value during the surveillance period. There were 45 of 62 such cases for seasonal H1N1, 25 of 36 for H3N2, and 82 of 97 for pH1N1, respectively.

For 2007‐2008 season (seasonal H1N1), we found that the mean viral AUC (±SD) among younger cases (≤15 years) was greater than in older cases (≥16 years) (6.62±0.79 vs 5.86±0.76 log_10_ copies/mL‐days, *P*<.01) (Table 3) in the univariate analysis. All other variables—sex (*P*=.11), comorbidity (*P*=.17), vaccination status (*P*=.18), and asymptomatic status (*P*=.17)—were entered into multivariate analysis model. The final model from multivariate analysis suggested that younger age (β, 0.75; 95% CI, 0.34 to 1.16; *P* <.01) was associated with an increased viral AUC for seasonal H1N1 (Table 4).

For 2008‐2009 season (H3N2), we found that the mean viral AUC (±SD) among younger cases (≤15 years) was greater than in older cases (≥16 years) (6.32±0.82 vs 5.45±0.72 log_10_ copies/mL‐days, *P*<.01) (Table 3) in the univariate analysis. Age was the only variable entered into multivariate analysis. The final model from multivariate analysis suggested that younger age (β, 0.87; 95% CI, 0.26 to 1.48; *P* =.02) was associated with an increased viral AUC for H3N2 (Table 4).

For 2009‐2010 season (pH1N1 2009), we found that the mean viral AUC (±SD) among younger cases (≤15 years) was significantly greater than in older cases (≥16 years) (6.24±0.68 vs 5.61±0.43 log_10_ copies/mL‐days, *P*<.01); the mean viral AUC among males was significantly greater than in females (6.28±0.53 vs 5.95±0.75 log_10_ copies/mL‐days, *P*=.02); and the mean viral AUC among symptomatic participants was significantly greater than in asymptomatic participants (6.15±0.63 vs 5.64±0.94 log_10_ copies/mL‐days, *P*=.04) (Table 3) in the univariate analysis. These three variables entered into multivariate analysis models. The final multivariable model showed that younger age (β, 0.62; 95% CI, 0.31 to 0.92; *P*<.01) and being symptomatic (β, 0.55; 95% CI, 0.11 to 0.98; *P*=.02) were associated with an increased viral AUC for pH1N1 (Table 4).

## 4 Discussion

We found that predictor variables varied by influenza A subtype. For seasonal H1N1, younger age was associated with a larger AUC, while female sex was associated with decreased peak viral load and reduced viral shedding duration while the presence of comorbidity was associated with increased peak viral load. For H3N2, younger age was associated with increased peak viral load and increased AUC. For pandemic H1N1, younger age was associated with increased peak viral load and increased viral AUC, female sex was associated with reduced peak viral load, while inapparent infection was associated with reduced peak viral load, reduced viral shedding duration, and reduced viral AUC.

Age was significantly associated with viral AUC for all the three subtypes of influenza A virus. Our analyses suggested younger cases had significantly higher viral AUC for all the three influenza A subtypes. As no data are available with respect to influenza viral AUC, our findings contribute to the literature.

Our results demonstrate that H1N1 viral shedding in males tends to reach higher levels and lasts longer than in females. One possible explanation is that females typically generate higher innate and adaptive immune responses accelerating viral clearance and reducing virus load.^22^, ^23^ However, our findings were not consistent for all three subtypes of influenza A virus, and further investigations to explain this discrepancy are needed.^24^

Comorbidity significantly increased peak viral load for H1N1, but not for pH1N1. For pH1N1, Lee et al.^25^also reported that comorbidity was not significantly associated with viral loads. We were unable to assess comorbidity associations for H3N2 from our data set, as the sample size was too small. However, Lee et al.^25^ suggested that comorbidity was significantly associated with viral loads for hospitalized H3N2 cases. Another study^15^ also suggested that the presence of major comorbidities was a significant factor that affected initial viral load in adult patients hospitalized with H3N2.

Asymptomatic status was a significant variable in all the three analyses for pH1N1, but not for seasonal H1N1. We were unable to assess association for H3N2 from our data set, as we did not detect any asymptomatic H3N2 cases in our study. Although it has been reported that asymptomatic shedding is common for both seasonal influenza A^26^ and pandemic strain,^10^ there are limited data that test the association between asymptomatic status and viral shedding patterns. Suess et al. reported that the proportion of asymptomatic cases was 18% for seasonal H3N2, 12% for pH1N1, and 14% for all influenza subtypes combined.^14^ The amount of viral shedding was similar between asymptomatic cases and symptomatic cases on days from the initial shedding; however, asymptomatic cases had shorter shedding duration for all influenza subtypes combined.^14^ We found a similar proportion of asymptomatic cases for pH1N1 (12%) as observed in the Suess study. However, we cannot compare our results directly to the Suess study with respect to the amount of virus shed and duration of viral shedding as the composition of asymptomatic influenza cases in our study differs from the Suess study. Considering the small number of asymptomatic cases in both Suess study (ie, 0 for seasonal H1N1, 2 of 11 cases for H3N2, 3 of 26 for pH1N1, and 4 of 22 for influenza B) and our study (ie, 9 of 62 for seasonal H1N1, 0 for seasonal H3N2, 12 of 97 for pH1N1), more research is needed to understand the association between asymptomatic status and viral shedding patterns.

We also found that vaccination (ie, trivalent inactivated influenza vaccine [TIV]) was associated with a reduced risk for H3N2 infection in 2008‐09 season, but increased the risk of pH1N1 in 2009‐10. There were good matches between the vaccines and the circulating viruses in both 2007‐2008 and 2008‐2009 seasons for seasonal influenza A. In 2007‐2008, the influenza A viruses circulating and included in the vaccine were A/Solomon Islands/03/2006 (H1N1)‐like virus and A/Wisconsin/67/2005 (H3N2)‐like virus;^27^ in 2008‐2009, the influenza A viruses circulating and included in the vaccine were A/Brisbane/59/2007[H1N1]‐like virus and A/Brisbane/10/2007[H3N2]‐like virus;^28^ 2009‐2010 season was dominated by pandemic H1N1.^29^ The reduced risk associated with vaccination in 2008‐09 season could be explained by the good match between influenza vaccine antigens and circulating strains in this season.^28^ However, there has been controversy about an increased risk of pH1N1 associated with seasonal influenza (TIV) vaccination. Two studies from Canada^29^, ^30^ reported an increased risk of pH1N1 associated with receipt of seasonal TIV. Conversely, there also were studies suggesting cross‐protection of TIV against pH1N1.^31^ A systematic review and meta‐analysis by Yin et al.^32^ showed that moderate cross‐protection might exist with no overall increased risk of infection from TIV.^32^ Our finding of pH1N1 risk associated with TIV is consistent with other findings in Canada.^30^

Strengths of this study include assessment over multiple seasons, inclusion of both adults and children, analysis by influenza A subtypes, active follow‐up for a relatively long period, and systematic swabbing of asymptomatic participants. One limitation is that we were not able to collect specimens daily from some participants (46%). Therefore, the value of peak viral load for these participants might not be their real peak value. Another limitation is that we did not assess VL for influenza B. Lastly, we conducted the study in Hutterite communities, which might limit generalizability of our results.

We conclude that patterns of influenza A molecular viral shedding vary by age, sex, comorbidity, and the presence of symptoms and predictor variables vary by influenza A subtype. The findings may inform interpretation of epidemiologic patterns of influenza for different influenza A subtypes.
